# Supplementary material for: Predicting orientation-dependent plastic susceptibility from static structure in amorphous solids via deep learning
Source: Nat Commun. 2021 Mar 8;12:1506. doi: 10.1038/s41467-021-21806-z (PMC7940643; doi:10.1038/s41467-021-21806-z)
Supplement: Supplementary file 1 — Supplementary Information [file 41467_2021_21806_MOESM1_ESM.pdf]

Supplementary Information for

Predicting orientation-dependent plastic susceptibility from static structure in amorphous solids via deep learning

Zhao Fan and Evan Ma

Department of Materials Science and Engineering, Johns Hopkins University, Baltimore, MD 21218, United States

Supplementary Notes

Supplementary Note 1: The optimization of r_c and Δ for the input features to CNN

During the optimization of CNN models, for 2D glasses we also tried r_c = 10.0 σ and Δ = 0.2 σ, or r_c = 5.0 σ and Δ = 0.1 σ with CNN containing 30 convolutional layers. We found that the accuracy can increase from 93.16% to 94.28% when using r_c = 10.0 σ, Δ = 0.2 σ, 30 convolutional layers compared to r_c = 5.0 σ, Δ = 0.2 σ, 25 convolutional layers, see Supplementary Fig. 1**a**. And it is very likely that the accuracy would be higher if there were more training data because the power of deeper CNN models has to be leveraged with larger training data and, as seen from Supplementary Fig. 23**a**, validation accuracy did not flatten until the training dataset size increases to ~19.6 million when training a CNN model containing 25 convolutional layers. (Note that for 2D glasses, we did not try 30 convolutional layers for f_{thres} = 0.5%, because the accuracy achieved with 25 convolutional layer is already close to the corresponding ceiling and our training dataset size was only 1.96 million when f_{thres} = 0.5%, which will not leverage the power of deeper CNN models). For 3D glasses, we tried r_c =12.5 Å and Δ=0.5 Å with CNN containing 25 convolutional layers (see Supplementary Fig. 1**b**), and the possible reasons why it did not improve accuracy significantly are that the current dataset is not big enough and/or a very small mini-batch size of 32 was used due to limited computer memory. These suggest that the accuracy, especially for 3D glasses, can be further improved if more training data and memory are available. However, doubling our current datasets demands huge computation resources. We project that with ever increasing training dataset size the accuracy may approach the upper bound. An example along this line was ResNet[1], which contains more than 100 convolutional layers.

Supplementary Note 2: The importance of including medium range structural information

We also tried to reduce r_c from its optimized value of 5.0 σ (9.6 Å) for the 2D (3D) glass, while keeping the same CNN architecture and the same ratio of $2r_c/\Delta$. As shown in Supplementary Fig. 1, the validation accuracy decreases gradually. This demonstrates the importance of including surrounding particles beyond the first nearest neighbor shell in predicting plastic susceptibility, and in particular the environment in the medium range on the order of 1 nm.

Supplementary Note 3: The choice of f_{thres}

We chose f_{thres} = 0.5%, because a previous study[2] found that a higher predictive accuracy can be achieved with a higher D^2_{min} threshold. We confirmed this in Supplementary Fig. 14. The trend for CNN and GNN cannot reflect fully their dependency of accuracy on f_{thres}, because large training datasets (see Supplementary Fig. 23) are needed. Here smaller f_{thres} also means smaller training datasets for the same number of glass samples. If the training dataset size is the same for different f_{thres}, the accuracy at small f_{thres} is expected to be higher.

Supplementary Note 4: Training all species together versus training each species separately

We found that training each species separately could not improve significantly the accuracy achieved for our CNN model, see Supplementary Fig. 15. Although training single species seems more effective, it also means smaller dataset size. In addition, training all species together in a single model is also easier to implement and use.

Supplementary Note 5: Particle-level prediction results of a given glass under different loading protocols by the three machine learning methods

Supplementary Fig. 16**a-d** show the spatial distribution maps of the CNN prediction for a given 2D glass, each map for one of the four different loading protocols, all at shear strain of 3.0%. We contrast these with the corresponding predictions from GNN (Supplementary Fig. 16**e-h**) and SVM (Supplementary Fig. 16**i-l**) models. It is important to observe from Supplementary Fig. 16 that our CNN model provide different predictions when the loading direction changes, and almost all of the particles with top 0.5% D^2_{min} are predicted correctly (red circles on the maps) and very few are predicted incorrectly (blue circles). In other words, almost all true fertile sites are recognized. There are some infertile particles mislabeled as having top 0.5% D^2_{min} (green circles on the maps). For the 2D glass, the GNN prediction is similar to CNN. In stark contrast, the SVM gives identical prediction for different loading protocols; as a result, too many particles are falsely predicted as belonging to the top 0.5% D^2_{min} group (green circles on the maps). There are also some fertile sites that are predicted incorrectly (blue circles).

Supplementary Note 6: Rotating and replicating 3D configurations for shearing along desired loading directions

In the LAMMPS package, the configurations with periodic boundary conditions in all three directions have to be cuboids, of which all the edges are parallel to the axes of the coordinate system. Thus, for the loading orientations of #7-24 listed in Supplementary Table 1, after appropriate rotation such that the shear direction and the normal direction of the shear plane are parallel respectively to X and Y axes of the coordinate system, we have to conduct appropriate replication to perform subsequent deformation simulations. Here we give an example to illustrate our specific operations. As shown in Supplementary Fig. 20, we first rotate the configurations such that [1,1,0] and [$\bar{1}$,1,0] are parallel to the X and Y axes, respectively, of the coordinate system, and then divide the configuration into four regions (Regions 1, 2, 3 and 4, as illustrated in Supplementary Fig. 20) and replicate atoms in each region and move these replicated atoms to the corresponding regions i, ii, iii and iv, respectively. This led us to a cuboid cell containing 64,000 atoms, with all edges parallel to [1,1,0], [$\bar{1}$,1,0] or [0, 0,1]. Finally, we can shear the box in the desired direction (the orientations of #7-12) after appropriate rotation. Similarly, one can rotate the configurations such that [1,0,1] and [$\bar{1}$,0,1] ([0,1,1] and [0,$\bar{1}$,1]) are parallel to X and Y axes, respectively, and conduct similar replications and rotations to shear samples along the loading orientations of #13-18 (#19-24). For all these shear simulations of cuboidal box containing 64,000 atoms, we have confirmed that the $D^2_{\min}$ of the replicated atoms is practically the same as that of the original atoms and we select atoms solely from the original atoms to construct training/validating/test datasets.

Supplementary Figures

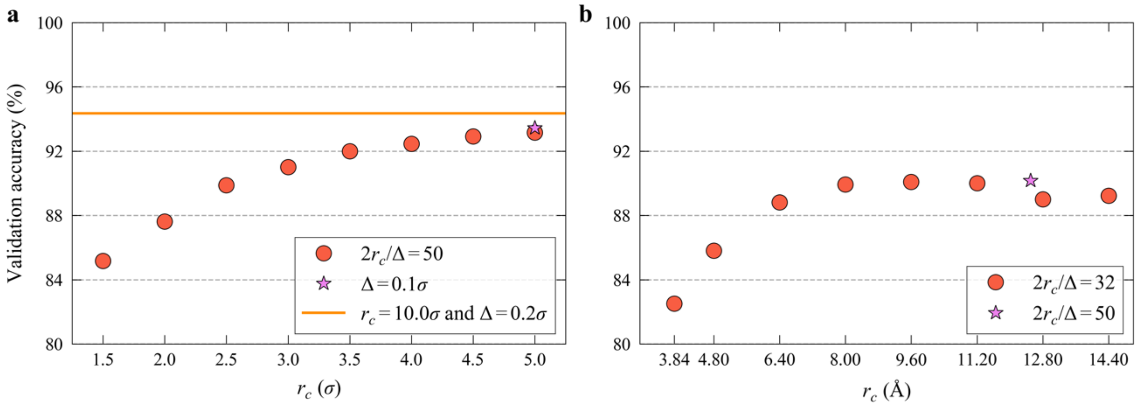

Supplementary Fig. 1. Effect of cutoff (r_c) and grid size (Δ) on validation accuracy. (**a**) 2D glasses at shear strain of 3.0% with f_{thres} of 5.0%. When r_c =10.0 σ or Δ=0.1 σ, the input images will have 100×100 pixels and thus the corresponding CNN model contains 30 convolutional layers. (**b**) 3D glasses at shear strain of 5.0% with f_{thres} of 0.5%. When $2r_c/\Delta$= 50, the input images will have 50×50×50 pixels and thus the corresponding CNN model contains 25 convolutional layers.

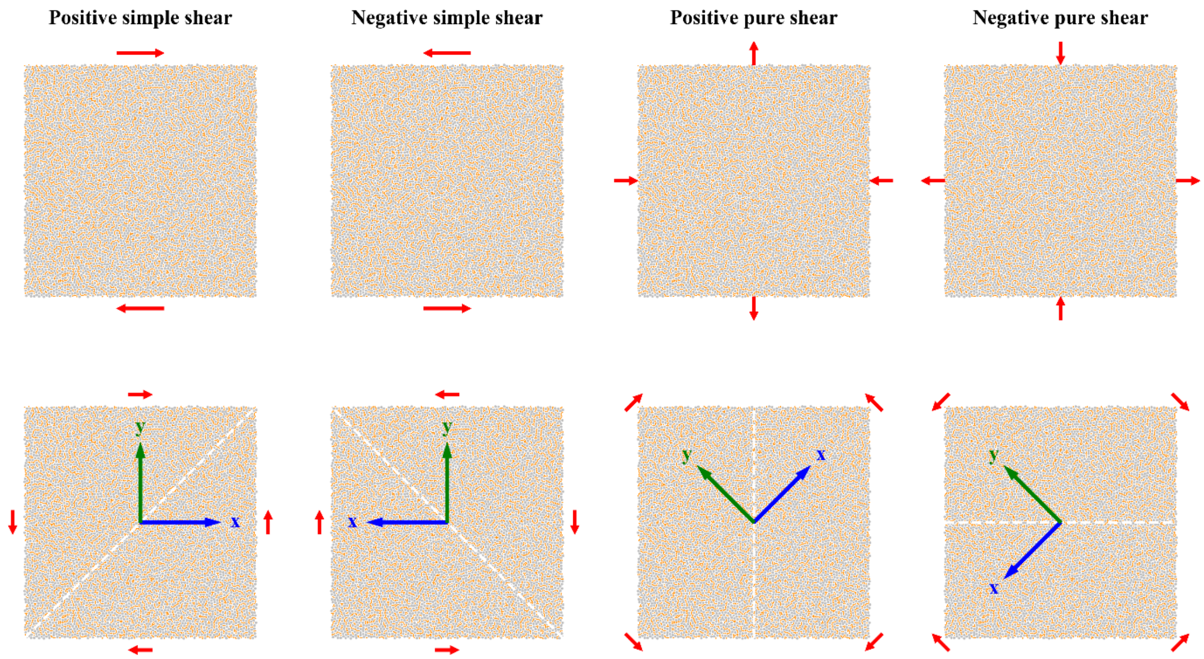

Supplementary Fig. 2. Deformation protocols for 2D model glasses. Upper panel: four different loading protocols in simulations; for each case the corresponding effective shear orientation is shown in the lower panel, with an appropriate coordinate system set up to compute the SDM. White dash line marks the macroscopic elongation direction.

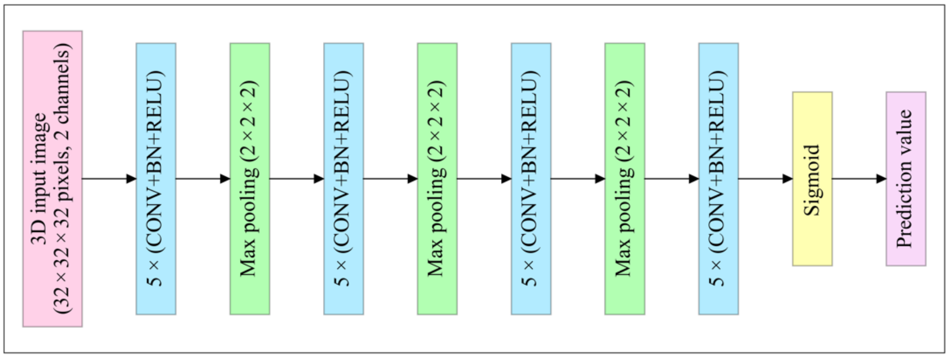

Supplementary Fig. 3. The CNN architecture for 3D glasses. The convolutional neural network (CNN) model for the 3D model glasses contains 20 3D convolutional (conv.) layers. A 3D max-pooling layer (green box) is inserted after every 5 convolutional layers. The last convolutional layer is followed by the output layer which is a sigmoid neuron.

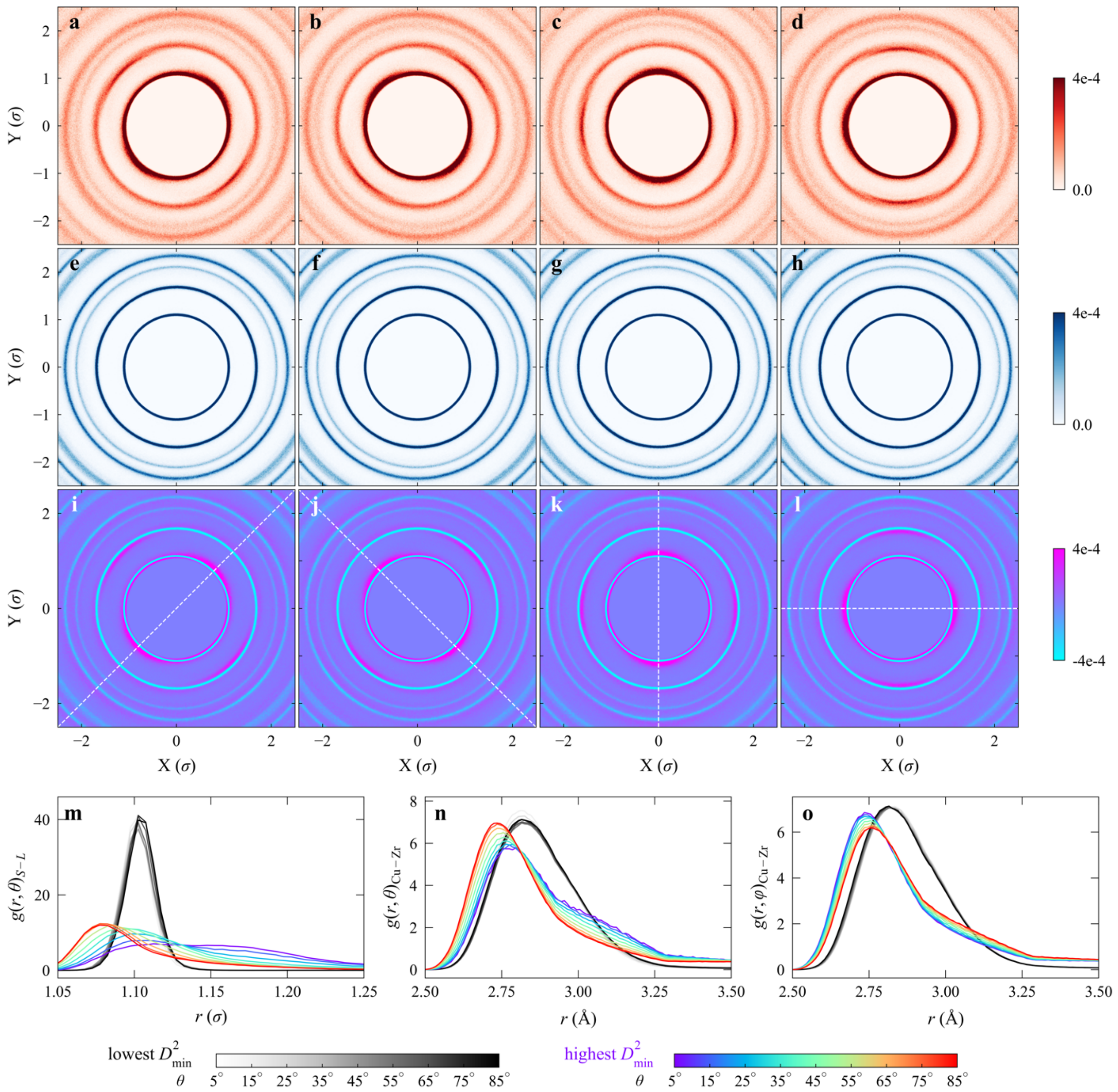

Supplementary Fig. 4. The difference in spatial distribution of surrounding *L* particles between *S* particles with the highest and those with the lowest plastic susceptibility. Each map in (**a-h**) shows the average SDM of the *L* particles in the neighborhood up to medium range for all *S* particles that have been identified to have large or small D^2_{min} when sheared to 3.0% strain, in 5,000 2D samples, for one of the four different loading protocols (from left to right: positive simple shear, negative simple shear, positive pure shear and negative pure shear). The SDM was calculated for these particles in the initial undeformed configurations. (**a-d**) are for central particles with top 0.5% of D^2_{min} while (**e-h**) for those with the lowest 0.5% of D^2_{min}. (**i-l**) show the difference in SDMs, which is calculated using the first row (**a-d**) *minus* the second row (**e-h**). The white dashed line in (**i-l**) represents the macroscopic elongation direction, $\boldsymbol{\zeta}$. (**m**) shows orientational partial (*S*-centered) pair correlation function $g_{\zeta}(r,\theta)$ for *S*-*L* correlation. Each $g_{\zeta}(r,\theta)$ curve is an average, over all samples and all loading orientations. For the 3D $Cu_{50}Zr_{50}$ glass, we show two orientational pair correlation functions for atoms that showed extreme D^2_{min} upon straining to 5%. (**n**) and (**o**) show Cu-centric $g_{\zeta}(r,\theta)$ and $g_{\zeta}(r,\varphi)$ curves, respectively, for Cu-Zr correlation.

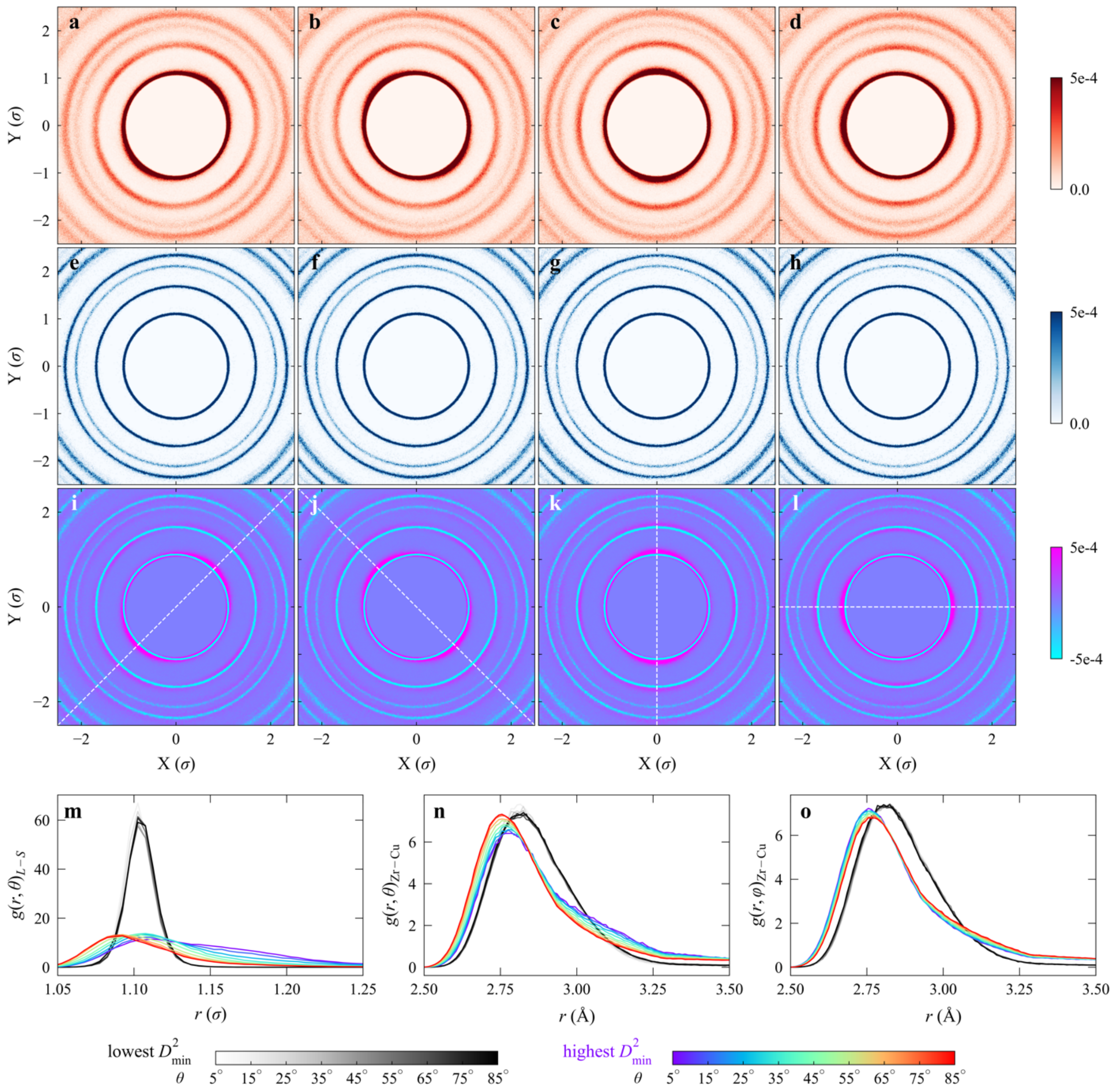

Supplementary Fig. 5. The difference in spatial distribution of surrounding *S* particles between *L* particles with the highest and those with the lowest plastic susceptibility. Each map in (**a-h**) shows the average SDM of the *S* particles in the neighborhood up to medium range for all *L* particles that have been identified to have large or small D^2_{min} when sheared to 3.0% strain, in 5,000 2D samples, for one of the four different loading protocols (from left to right: positive simple shear, negative simple shear, positive pure shear and negative pure shear). The SDM was calculated for these particles in the initial undeformed configurations. (**a-d**) are for central particles with top 0.5% of D^2_{min} while (**e-h**) for those with the lowest 0.5% of D^2_{min}. (**i-l**) show the difference in SDMs, which is calculated using the first row (**a-d**) *minus* the second row (**e-h**). The white dashed line in (**i-l**) represents the macroscopic elongation direction, $\boldsymbol{\zeta}$. (**m**) shows orientational partial (*L*-centered) pair correlation function $g_\zeta(r,\theta)$ for *L*-*S* correlation. Each $g_\zeta(r,\theta)$ curve is an average, over all samples and all loading orientations. For the 3D $Cu_{50}Zr_{50}$ glass, we show two orientational pair correlation functions for atoms that showed extreme D^2_{min} upon straining to 5%. (**n**) and (**o**) show Zr-centric $g_\zeta(r,\theta)$ and $g_\zeta(r,\varphi)$ curves, respectively, for Zr-Cu correlation.

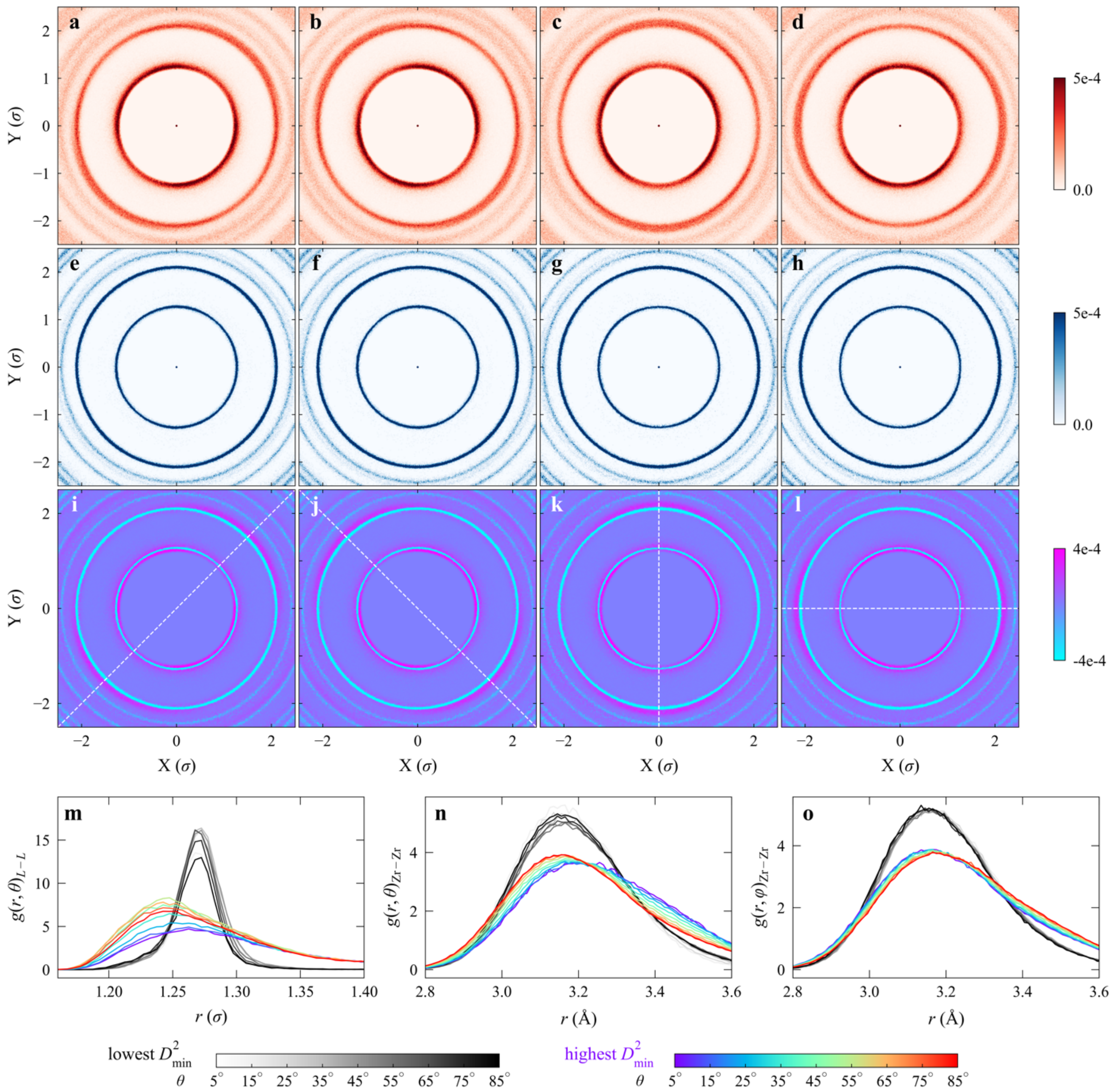

Supplementary Fig. 6. The difference in spatial distribution of surrounding *L* particles between *L* particles with the highest and those with the lowest plastic susceptibility. Each map in (**a-h**) shows the average SDM of the *L* particles in the neighborhood up to medium range for all *L* particles that have been identified to have large or small D^2_{min} when sheared to 3.0% strain, in 5,000 2D samples, for one of the four different loading protocols (from left to right: positive simple shear, negative simple shear, positive pure shear and negative pure shear). The SDM was calculated for these particles in the initial undeformed configurations. (**a-d**) are for central particles with top 0.5% of D^2_{min} while (**e-h**) for those with the lowest 0.5% of D^2_{min}. (**i-l**) show the difference in SDMs, which is calculated using the first row (**a-d**) *minus* the second row (**e-h**). The white dashed line in (**i-l**) represents the macroscopic elongation direction, $\boldsymbol{\zeta}$. (**m**) shows orientational partial (*L*-centered) pair correlation function $g_{\zeta}(r,\theta)$ for *L*-*L* correlation. Each $g_{\zeta}(r,\theta)$ curve is an average, over all samples and all loading orientations. The distribution of *L* neighbors changes with θ mainly in the peak intensity, also indicating the asymmetric packing. For the 3D $Cu_{50}Zr_{50}$ glass, we show two orientational pair correlation functions for atoms that showed extreme D^2_{min} upon straining to 5%. (**n**) and (**o**) show Zr-centric $g_{\zeta}(r,\theta)$ and $g_{\zeta}(r,\varphi)$ curves, respectively, for Zr-Zr correlation.

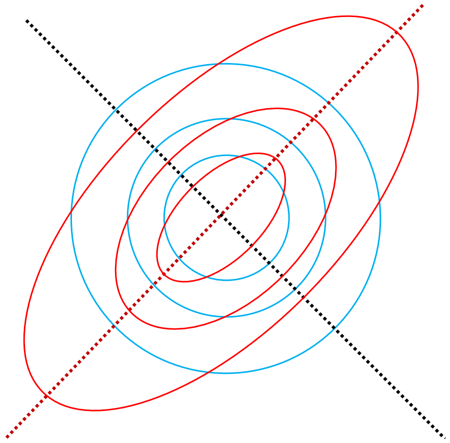

Supplementary Fig. 7. Schematic illustrating the critical structural differences that influence the local mechanical response and its anisotropy. This insight is first revealed from the difference in SDMs between particles with the highest versus those with the lowest plastic susceptibility in 2D glasses and then further confirmed for both 2D and 3D glasses through contrasting orientational pair correlation functions, see Fig. 2 and Supplementary Figs. 4-6. Specifically, for particles with low plastic susceptibility, their neighbors tend to form circularly or spherically symmetric shells with almost uniform packing intensity, as schematically idealized using the blue circles in the schematic above. In contrast, packing tends to be more asymmetric surrounding fertile sites: the neighboring shells are more like ellipses, shown as the red elliptical circles, each with non-uniform particle distribution. For a fertile site to be activated upon a particular mechanical loading, the longest and shortest axes of neighboring shells/ellipses should be parallel to the elongation (the red dotted line) and contraction (the black dotted line) directions imposed by the loading, respectively. In other words, a fertile site is the most prone to be activated when its surrounding packing along the elongation direction is the loosest and the packing along the contraction direction is the densest.

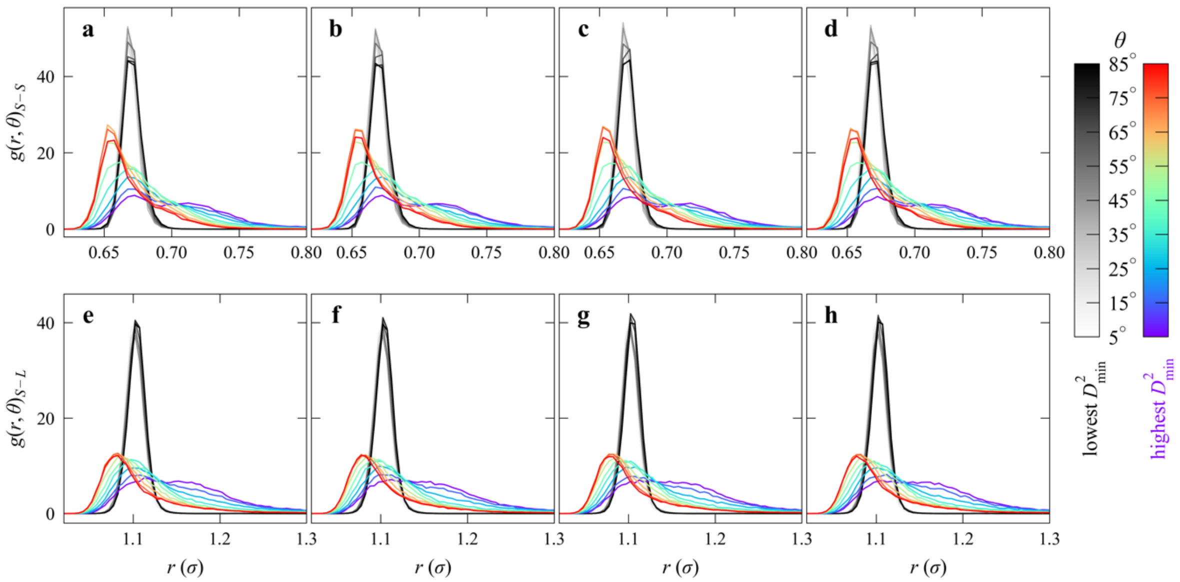

Supplementary Fig. 8. The orientational partial pair correlation function $g_\zeta(r,\theta)$ of S particles that showed the top 0.5% $D^2{}_{min}$ after being shear strained to 3.0% (colored), compared with that for the lowest 0.5% $D^2{}_{min}$ (grey). The upper row is for the neighboring S particles, whereas the lower row is for L particles in the neighborhood. Each curve is the average over all surrounding S (or L) particles from all 5,000 samples. From left to right the columns correspond to positive simple shear, negative simple shear, positive pure shear and negative pure shear, respectively. The (**a**-**d**) are similar to each other and are averaged into Fig. 2**m** in the main text. And (**e**-**h**) are similar to each other and are averaged into Supplementary Fig. 4**m**.

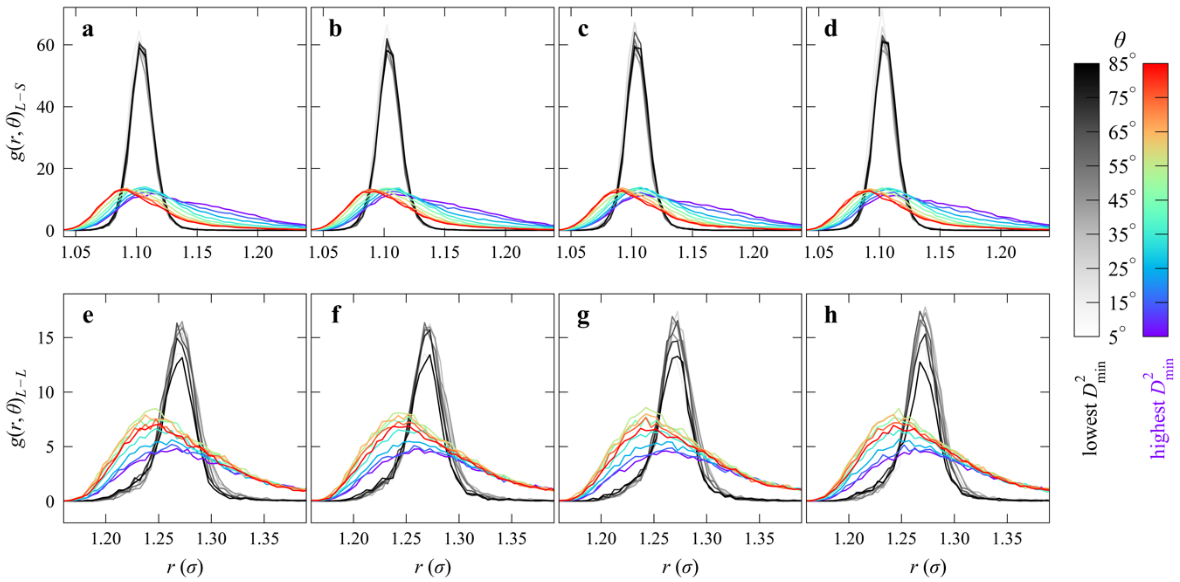

Supplementary Fig. 9. The orientational partial pair correlation function $g_\zeta(r,\theta)$ of L particles that showed the top 0.5% $D^2{}_{\mathrm{min}}$ after being shear strained to 3.0% (colored), compared with that for the lowest 0.5% $D^2{}_{\mathrm{min}}$ (grey). The upper row is for the neighboring S particles, whereas the lower row is for L particles in the neighborhood. Each curve is the average over all surrounding S (or L) particles from all 5,000 samples. From left to right the columns correspond to positive simple shear, negative simple shear, positive pure shear and negative pure shear, respectively. The (**a-d**) are similar to each other and are averaged into Supplementary Fig. 5**m**. And the (**e-h**) are similar to each other and are averaged into Supplementary Fig. 6**m**.

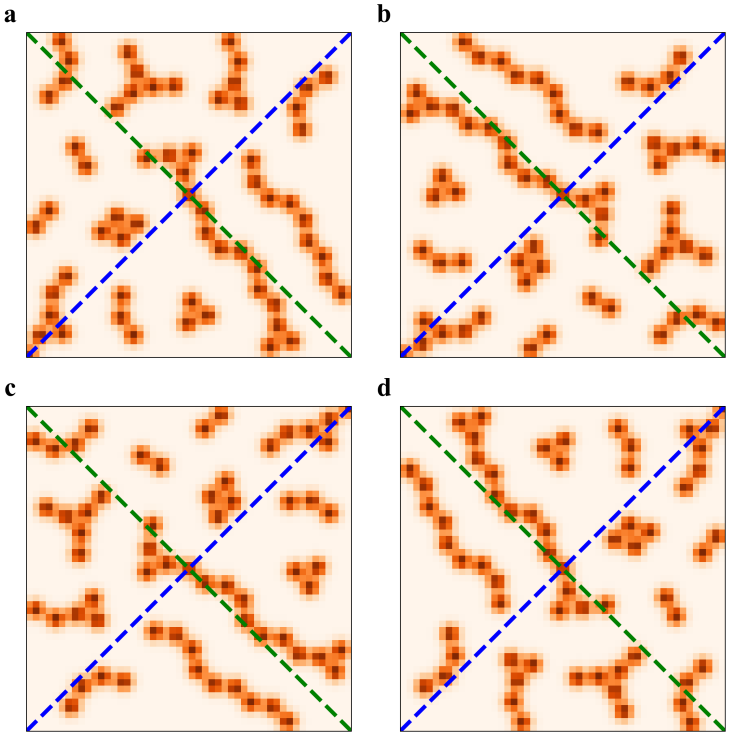

Supplementary Fig. 10. Equivalent local configurations based on mirror-symmetries. Here we use the SDMs of species S for a 2D local configuration to illustrate the mirror operations. (**a**) and (**b**) are mirrored to each other along macroscopic elongation direction (blue dash line) in the plane of $\boldsymbol{\iota} \times \boldsymbol{\eta}$; (**a**) and (**c**) are mirrored to each other along macroscopic contraction direction (green dash line); (**a**) and (**d**) will be the same after two mirror operations (along both elongation and contraction lines), which is equivalent to a rotation by 180° around the center of images. The 4 images can generate another 4 mirrored ones, across the plane of $\boldsymbol{\iota} \times \boldsymbol{\eta}$ for 3D configurations. These mirror operations will not change the packing density of local configurations along elongation or contraction orientations and thus those mirrored configurations are expected to exhibit similar mechanical response, which is confirmed in the Supplementary Fig. 11.

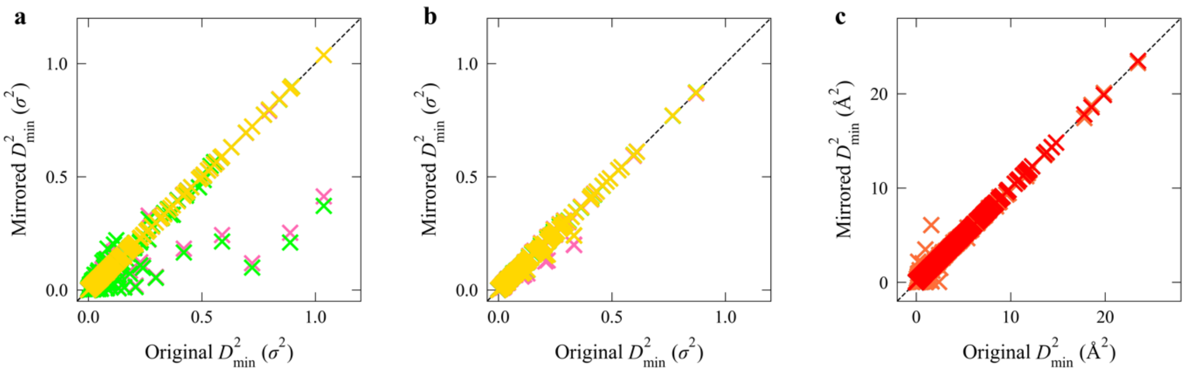

Supplementary Fig. 11. Mirrored samples show almost identical mechanical response. (**a**) and (**b**) are for a 2D glass strained to 2.5% in simple and pure shear, respectively. (**c**) is for 3D glasses strained to 5.0% in simple shear. Each symbol in the plots represents D^2_{min} of a single particle in one the of 3 (or 7) mirrored samples (see more details about mirror-symmetry in the Supplementary Fig. 10) against that in the original 2D (or 3D) glass. Almost all symbols locate on the diagonal dash lines, indicating that particles in mirrored samples show almost identical mechanical response as those in the original samples.

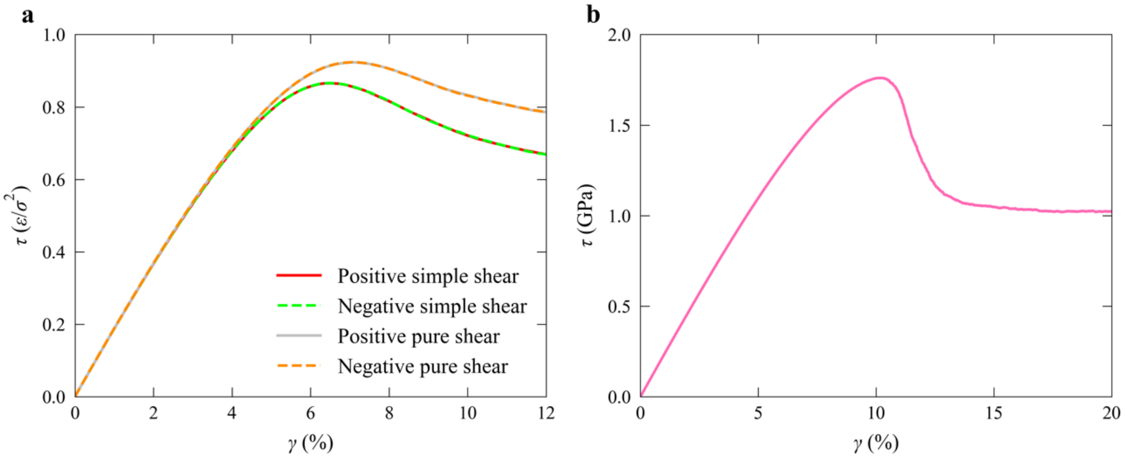

Supplementary Fig. 12. (**a**) The shear stress-strain curves for 2D glasses, averaged over 5,000 post-loading samples for each of the four different protocols. For simple shear, the shear strain $\gamma = |\gamma_{xy}|$, the shear stress $\tau = |\tau_{xy}|$, and for pure shear, $\gamma = |\epsilon_{xx} - \epsilon_{yy}|$, $\tau = \frac{|\sigma_{xx} - \sigma_{yy}|}{2}$. (**b**) The shear stress-strain curve for $Cu_{50}Zr_{50}$ 3D glasses, averaged over 20 samples sheared to large (up to 20%) strain. Each sample was simple-sheared in 24 different orientations and thus the curve is the average of 480 curves.

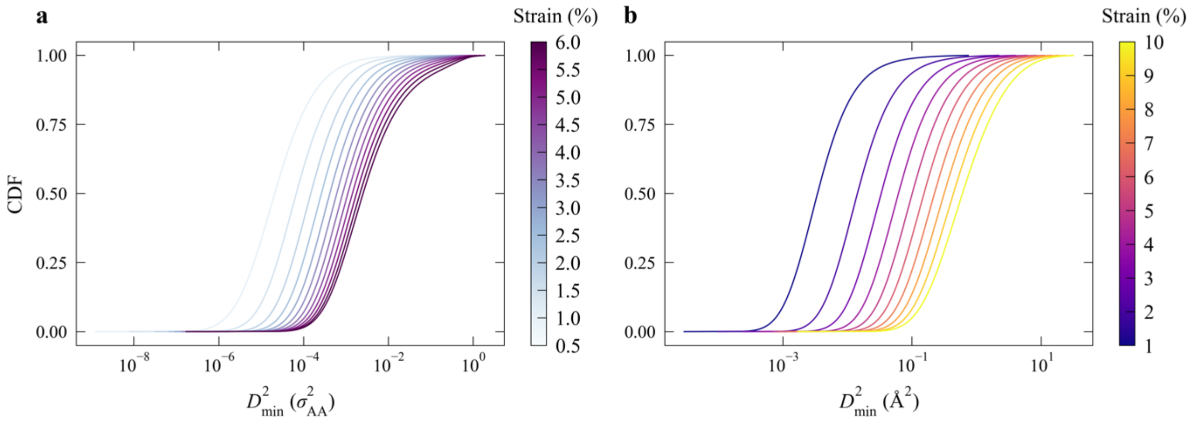

Supplementary Fig. 13. The cumulative distribution function (CDF) of D^2_{min} at different shear strains for the 2D glasses (**a**) and the 3D $Cu_{50}Zr_{50}$ glasses (**b**).

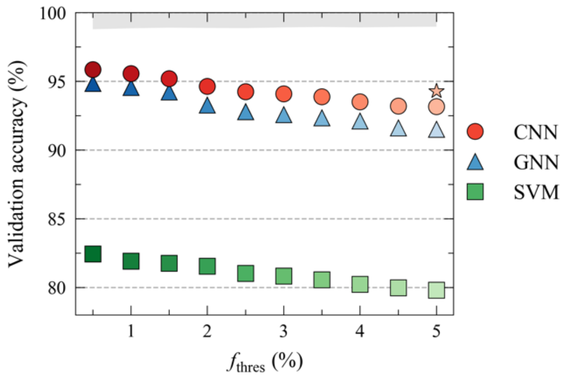

Supplementary Fig. 14. Validation accuracy versus f_{thres} for the 2D L-J glasses at a constant strain of 3.0%. The shaded region marks the upper bound. Note here that smaller f_{thres} means smaller training datasets for the same number of glass samples. Since both CNN and GNN require large training datasets (see Supplementary Fig. 23) to reach high accuracy, the accuracy at small f_{thres} is expected to be higher if the training dataset size was the same for different f_{thres}. The red star represents the accuracy of 94.28% achieved with a CNN model containing 30 convolutional layers on images with r_c =10.0 σ and Δ=0.2 σ. This example of successfully improved CNN accuracy shows again the advantage of CNN, further demonstrating its capability to excel over the GNN model (The CNN now performs considerably better when compared with the 91.52% achieved at the same f_{thres} with the GNN).

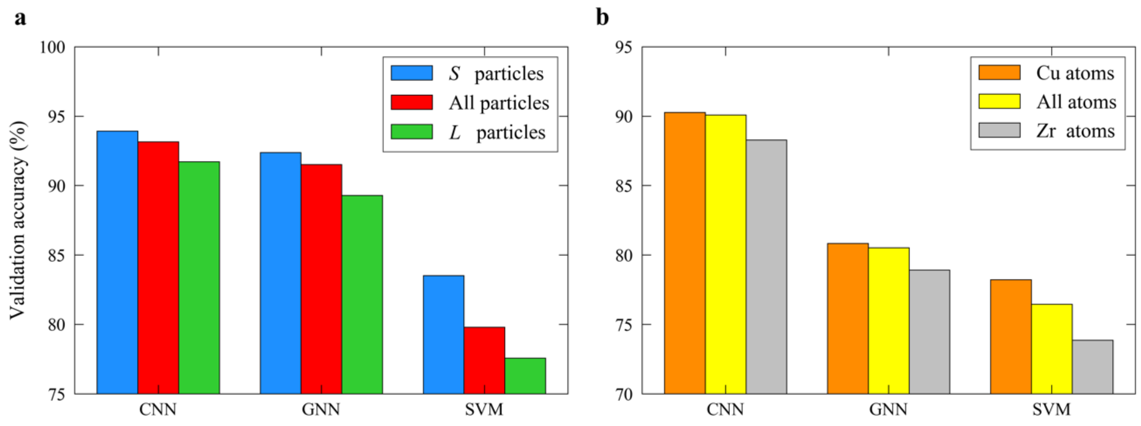

Supplementary Fig. 15. Effect on validation accuracy, comparing training each species separately versus training all species together. (**a**) 2D glasses at shear strain of 3.0% with f_{thres} of 5.0%. (**b**) 3D $Cu_{50}Zr_{50}$ glasses at shear strain of 5.0% with f_{thres} of 0.5%.

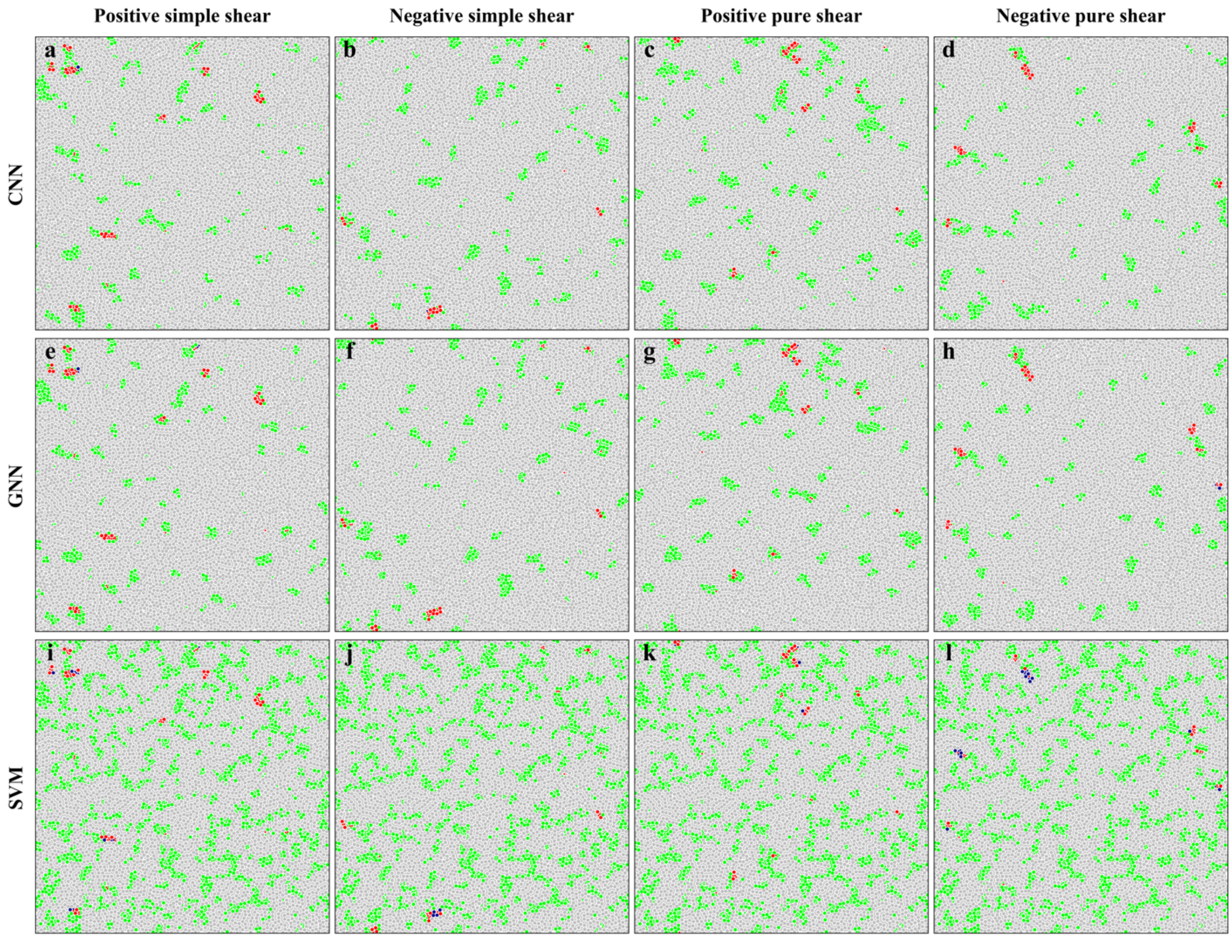

Supplementary Fig. 16. Spatial distribution map of prediction results for a given 2D glass sample. The particles with top 0.5% D^2_{min} are regarded as fertile sites after the model was loaded to shear strain of 3.0%. The results on the upper, middle and lower panels are from CNN, GNN, and SVM models, respectively. From left to right the columns represent four different loading protocols, i.e., positive simple shear, negative simple shear, positive pure shear and negative pure shear, all performed on the same glass. On the maps, fertile sites predicted correctly are marked with red color; non-fertile-sites predicted correctly are marked silver while those mislabeled as fertile are marked light green; fertile sites mislabeled as non-fertile are marked blue.

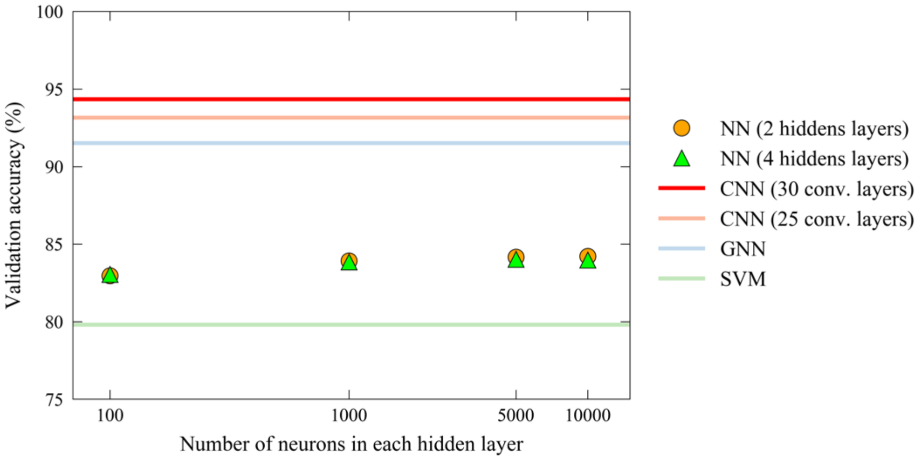

Supplementary Fig. 17. Comparing fully connected neural networks (NN) with CNN, GNN and SVM. This plot shows the validation accuracy (symbols) reached, on 2D glasses at strain 3.0% with f_{thres} = 5.0%, via the fully connected NNs with different architectures (each NN contains 2 or 4 hidden layers with different number of neurons and the number of neurons in different hidden layers is identical within a NN). For comparison, the accuracy achieved by using other machine learning methods is represented by lines with different colors (see legend). The input to these simple NNs is the same as the images fed to CNN, but flattened into vectors.

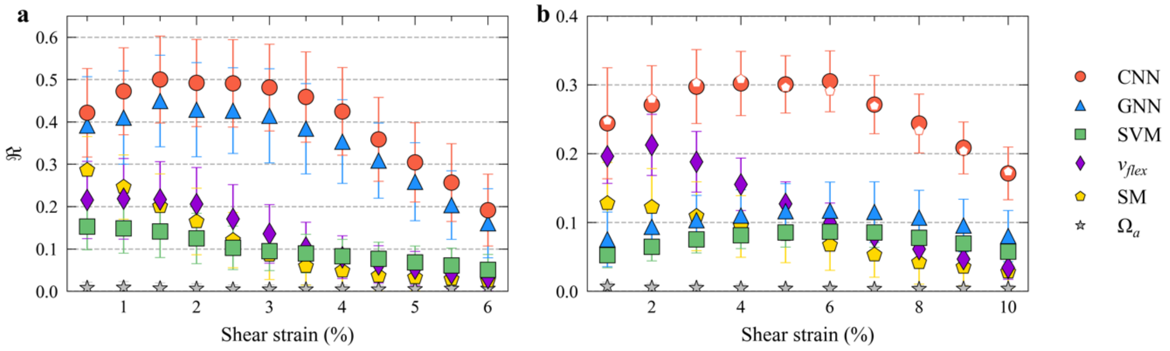

Supplementary Fig. 18. Correctly predicted fraction given by the three machine learning models and three physical-indicator-based methods (flexibility volume (v_{flex}), soft modes (SM), atomic volume (Ω_a)): (**a**) for 2D L-J and (**b**) for 3D $Cu_{50}Z_{50}$ glasses, respectively. Here the predictive capability is evaluated in term of $\mathcal{R}$, which is the overlap ratio given by the number of particles belonging simultaneously to two groups that are intersecting, divided by the number of particles known to have the top 0.5% D^2_{min}. In this context the "intersection" particles are those that belong not only to the group known to have the top 0.5% D^2_{min}, but at the same time also to the group having top 0.5% plastic susceptibility predicted using one of the six methods. As here we only allow a narrow bin of the top 0.5% D^2_{min}, this criterion is the most stringent, arguably to the extreme. The error bars correspond to standard deviation among 50 (10) different samples and 4 (24) different loading protocols for 2D (3D) glasses. For 3D glasses, all predictions used samples each containing 32,000 atoms, except that the soft mode analysis was conducted on 10 different samples each containing 10,000 atoms. For a fair comparison, the CNN analysis was also conducted on the smaller samples (open pentagons in (**b**)).

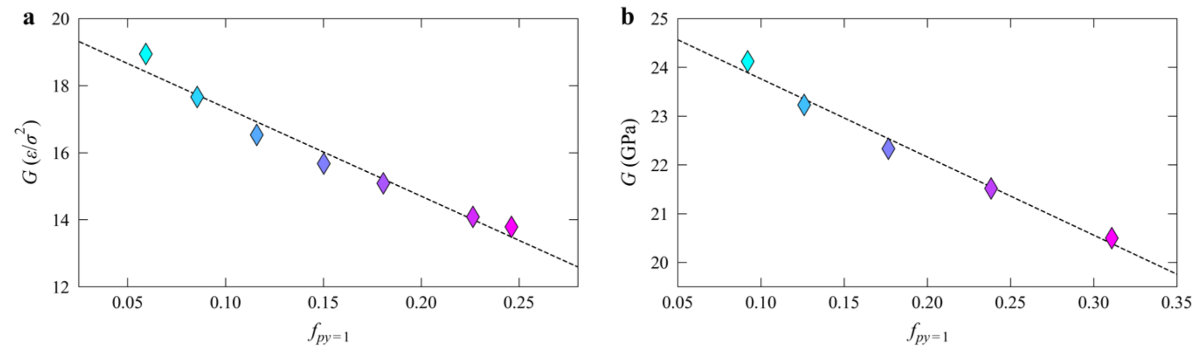

Supplementary Fig. 19. Shear modulus (G) versus the CNN-predicted fraction of particles with class probability > 0.5. This fraction is denoted $f_{py=1}$ and used as the x axis, for (**a**) 2D L-J and (**b**) 3D $Cu_{50}Zr_{50}$ glass samples with different processing histories. Each data point is averaged over 50 (10) samples across 4 (24) loading protocols for the 2D (3D) glasses, sheared to a strain of 1% (2%). The dashed line serves as a guide to the eye.

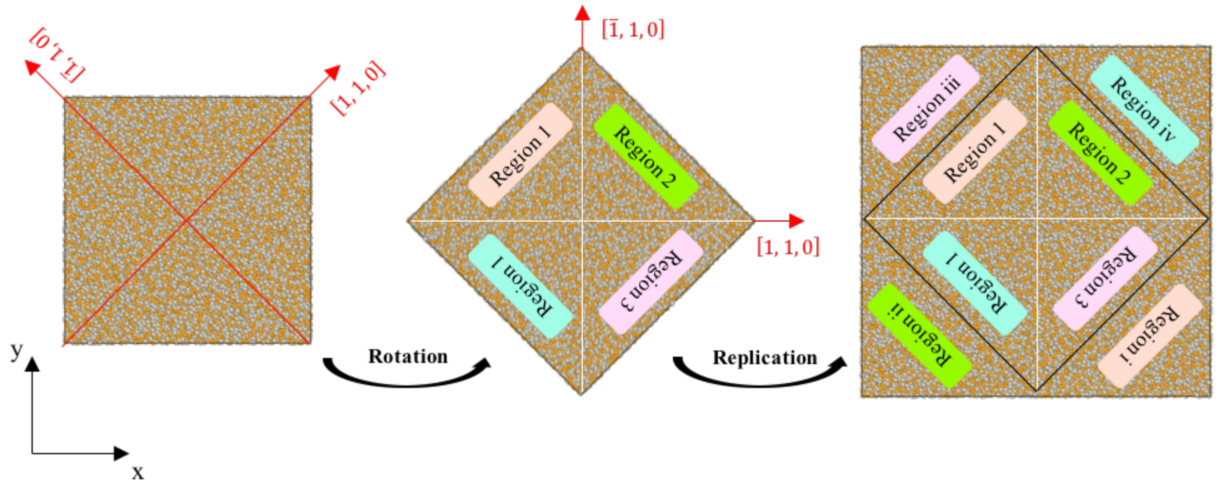

Supplementary Fig. 20. Schematic of rotating and replicating configurations in order to deform 3D metallic glasses along desired loading directions.

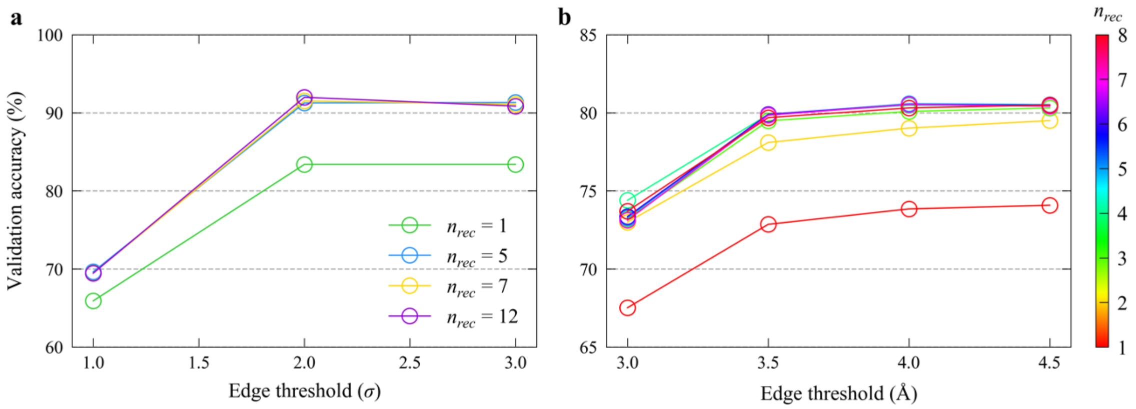

Supplementary Fig. 21. Accuracy achieved via the GNN model, when the hyperparameters are varied, for (**a**) 2D glasses at strain of 3.0% with f_{thres} of 5.0% and (**b**) 3D $Cu_{50}Zr_{50}$ glasses at strain of 5.0% with f_{thres} of 0.5%. The edge threshold is varied at a fixed number of unrolls of the network (n_{rec}), and the n_{rec} is varied at a fixed edge threshold.

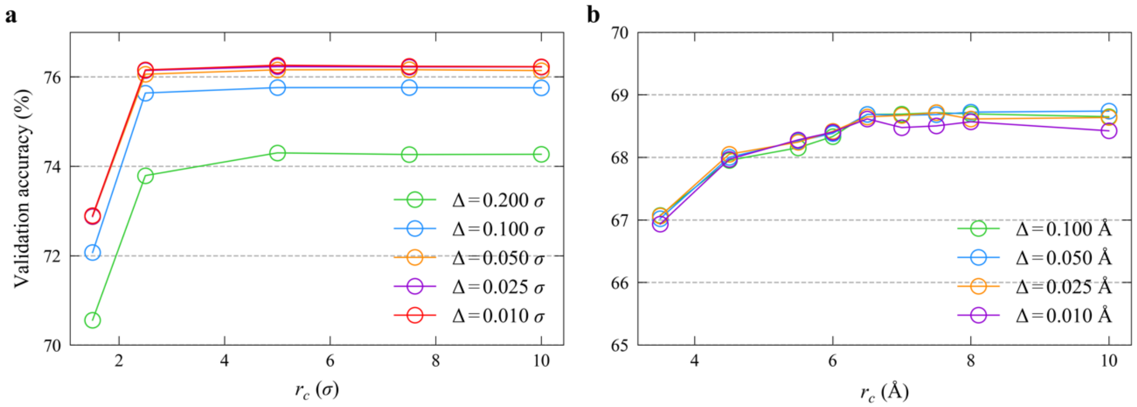

Supplementary Fig. 22. Accuracy reached via the SVM models versus r_c and Δ, for (**a**) 2D glasses at strain of 3.0% with f_{thres} of 5.0%, and (**b**) 3D $Cu_{50}Zr_{50}$ glasses at strain of 5.0% with f_{thres} of 0.5%. Here the training did not include angular structural functions.

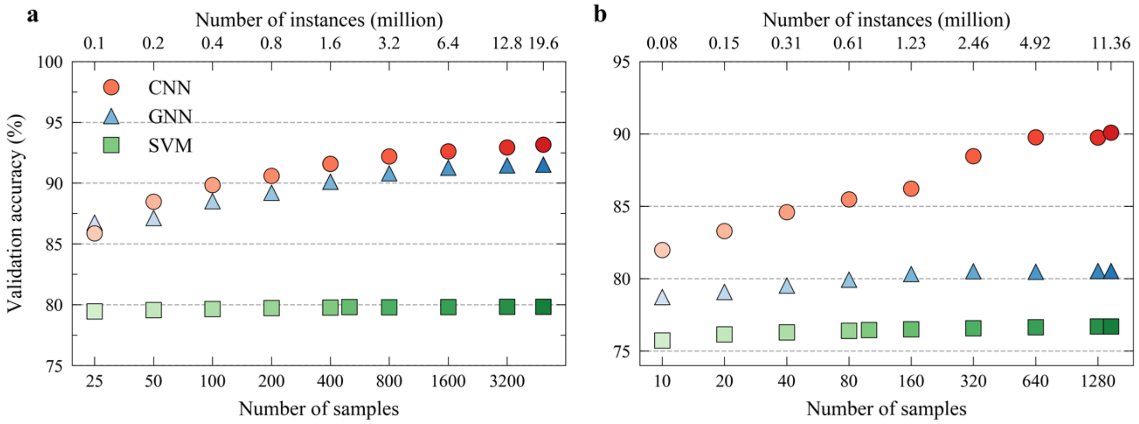

Supplementary Fig. 23. The effect of training dataset size on validation accuracy. (**a**) and (**b**) are for 2D glasses at shear strain of 3.0% with f_{thres} of 5.0% and 3D $Cu_{50}Zr_{50}$ glasses at shear strain of 5.0% with f_{thres} of 0.5%, respectively. The largest training dataset for the 2D glasses contains 4,900 samples each containing 10,000 particles and sheared in 4 loading orientations, so with f_{thres} of 5.0%, the largest dataset contained ~19.6 million instances. The largest training dataset for the 3D glasses contains 1,480 samples each with 32,000 atoms and sheared along 24 loading orientations, so with f_{thres} of 0.5%, the largest dataset contains ~11.36 million instances.

Supplementary Tables

Supplementary Table 1 The specific shear direction (***ι***) and the normal direction of the shear plane (***η***) for the 24 different loading orientations applied on 3D glasses.

#	shear direction (***ι***)	normal direction of shear plane (***η***)
1	[1, 0, 0]	[0, 1, 0]
2	[-1, 0, 0]	[0, 1, 0]
3	[1, 0, 0]	[0, 0, 1]
4	[-1, 0, 0]	[0, 0, 1]
5	[0, 1, 0]	[0, 0, 1]
6	[0, -1, 0]	[0, 0, 1]
7	[1, 1, 0]	[-1, 1, 0]
8	[-1, -1, 0]	[-1, 1, 0]
9	[1, 1 0]	[0, 0, 1]
10	[-1, -1, 0]	[0, 0, 1]
11	[-1, 1, 0]	[0, 0, 1]
12	[1, -1, 0]	[0, 0, 1]
13	[1, 0, 1]	[-1, 0, 1]
14	[-1, 0, -1]	[-1, 0, 1]
15	[1, 0, 1]	[0, 1, 0]
16	[-1, 0, -1]	[0, 1, 0]
17	[-1, 0, 1]	[0, 1, 0]
18	[1, 0, -1]	[0, 1, 0]
19	[0, 1, 1]	[0, -1, 1]
20	[0, -1, -1]	[0, -1, 1]
21	[0, 1, 1]	[1, 0, 0]
22	[0, -1, -1]	[1, 0, 0]
23	[0, -1, 1]	[1, 0, 0]
24	[0, 1, -1]	[1, 0, 0]

Supplementary Table 2 The number of input features and the number of trainable parameters in the various models for 2D L-J glasses.

Model	Number of input features	Number of trainable parameters
SVM	840	841
GNN	~330,000	70,657
CNN ($2r_c/\Delta$=50, 25 convolutional layers)	5,000	5,532,381
CNN ($2r_c/\Delta$=100, 30 convolutional layers)	20,000	22,131,101

Supplementary Table 3 The number of input features and the number of trainable parameters in the various models for 3D Cu-Zr glasses.

Model	Number of input features	Number of trainable parameters
SVM	720	721
GNN	~698,000	70,721
CNN ($2r_c/\Delta$=32, 20 convolutional layers)	65,536	1,853,701
CNN ($2r_c/\Delta$=50, 25 convolutional layers)	250,000	2,340,601

Supplementary References

1. He, K., Zhang, X., Ren, S. & Sun, J. Deep Residual Learning for Image Recognition. On pages 770–778 (2016).
2. Cubuk, E. D. *et al.* Identifying Structural Flow Defects in Disordered Solids Using Machine-Learning Methods. *Phys. Rev. Lett.* **114**, 108001 (2015).
